# Next generation models for storage and representation of microbial biological annotation

**DOI:** 10.1186/1471-2105-11-S6-S15

**Published:** 2010-10-07

**Authors:** Daniel J Quest, Miriam L Land, Thomas S Brettin, Robert W Cottingham

**Affiliations:** 1 Biosciences Division, Oak Ridge National Laboratory, P.O. Box 2008, Oak Ridge, TN 37831-6420, USA

## Abstract

**Background:**

Traditional genome annotation systems were developed in a very different computing era, one where the World Wide Web was just emerging.  Consequently, these systems are built as centralized black boxes focused on generating high quality annotation submissions to GenBank/EMBL supported by expert manual curation.  The exponential growth of sequence data drives a growing need for increasingly higher quality and automatically generated annotation.

Typical annotation pipelines utilize traditional database technologies, clustered computing resources, Perl, C, and UNIX file systems to process raw sequence data, identify genes, and predict and categorize gene function.  These technologies tightly couple the annotation software system to hardware and third party software (e.g. relational database systems and schemas).  This makes annotation systems hard to reproduce, inflexible to modification over time, difficult to assess, difficult to partition across multiple geographic sites, and difficult to understand for those who are not domain experts.  These systems are not readily open to scrutiny and therefore not scientifically tractable.

The advent of Semantic Web standards such as Resource Description Framework (RDF) and OWL Web Ontology Language (OWL) enables us to construct systems that address these challenges in a new comprehensive way.

**Results:**

Here, we develop a framework for linking traditional data to OWL-based ontologies in genome annotation.  We show how data standards can decouple hardware and third party software tools from annotation pipelines, thereby making annotation pipelines easier to reproduce and assess.  An illustrative example shows how TURTLE (Terse RDF Triple Language) can be used as a human readable, but also semantically-aware, equivalent to GenBank/EMBL files.

**Conclusions:**

The power of this approach lies in its ability to assemble annotation data from multiple databases across multiple locations into a representation that is understandable to researchers.  In this way, all researchers, experimental and computational, will more easily understand the informatics processes constructing genome annotation and ultimately be able to help improve the systems that produce them.

## Background

Genome annotation systems provide utilities to identify genes in a new genome sequence, classify each gene according to its most likely functions, and predict biochemical pathways that may exist in the sequenced organism.  These systems have provided the foundation for many bioinformatics processes and analyses, but an in-depth understanding of the algorithms, data processing procedures and hardware requirements utilized is available only to a select few systems maintainers in large genome centers [1].  The advent of next generation sequencing technologies enables individual labs to replicate the sequencing capacity traditionally found only at genome sequencing centers. The capacity to annotate and understand genome sequences (i.e. the sequence annotation information technology and infrastructure) is not easily transferred because of the hardware costs (e.g. clustered computers), the bioinformatics expertise required to integrate hundreds of tools, and the expertise of manual annotators.  As systems biology integrates more sources of information, the complexity of annotation will continue to increase.  It is unlikely that even large centres will be able to handle the scope and complexity of what annotation is likely to become, ultimately a computational model of the living cell.

Annotation systems such as RAST [2], the annotation services at JCVI [3],  have started to close this gap by providing annotation services online that are free and readily available to individual researchers.  However, it is still difficult to determine the quality, consistency, and interoperability of the outputs from these systems [5].

Technologies such as the Distributed Annotation System (DAS) [6,7] provide a good start on these challenges by providing an Extensible Markup Language (XML) [8] standard for the annotation data exchange.  This allows sharing data at the syntax level, however the data is not understandable to the machine at the semantic level. Semantic interoperability is critical when combining data streams from multiple sources, or when providing different and useful views on data.  Semantic interoperability is a critical first step in transforming data into usable knowledge.

Recent advances in unambiguous knowledge representation vocabularies (ontologies) provide promise for recording the underlying semantics in biological data.  Gene Ontology (GO) provides a controlled set of terms for gene products [9].  The Open Biological and Biomedical Ontologies initiative (OBO) [10] has been collecting a series of ontologies for biological and medical domains such as the Sequence Ontology [11], and Systems Biology Ontology [12]. The Comparative Data Analysis Ontology [13] provides a set of concepts for biological comparisons such as alignment and phylogentic trees.  The BioPAX ontology [14] provides a common framework for relating pathway datasets.  Future data source integration will require the ability to tie data and analysis to these common unambiguous biological concept frameworks. More importantly, providing data interoperability and additional utility requires the frameworks be leveraged and connected to data stores under a common standard. 

The OWL Web Ontology Language [15,16] and OWL 2 [17] are W3C recommended standards for the construction of the Semantic Web [18,19].  OWL allows data aggregation, integration and construction of a distributed system for automated biological knowledge discovery.  Protocols for sharing biological analysis, such as SSWAP [20], are emerging.  However, many biological ontologies predate the OWL standard.  Some converters exist for transforming ontologies to the OWL standard [21], however not all attributes are easily transferable.  Consequently, third party tools link to ontologies in unstructured ways.  There is no guarantee that the GO terms referenced are logically consistent with one another or that the reference is consistent with the programmatic model used in the annotation system or with the semantic model intended by the GO maintainers [22].  

In the long term, new annotation systems must be built that are more understandable, accessible, and modifiable by biological experts.  The new systems must decouple data from the hardware and algorithms used for analysis.  The first step in this process is robust standards (e.g. XML) for sharing information across systems.  The second step is the development of ontologies to share semantic meaning across systems.  Both are well underway.  The development of a framework for linking traditional data to OWL based ontologies within the context of genome annotation is the next step.  Described here is a proof-of-principle that accomplished this next step through an analysis of the data storage resources used in conventional annotation systems, prototyping wrappers that convert the inputs and outputs of algorithms into resource description framework statements (RDF), prototyping storage solutions to store and query RDF statement repositories, and prototyping the conversion process from RDF statements into traditional standards.

## Results

### Overview

**Figure 1:**
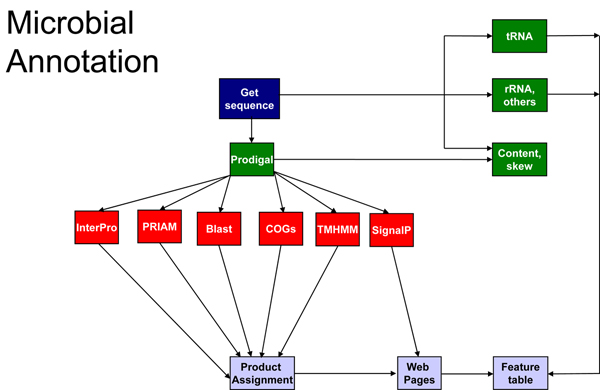
An overview of the Oak Ridge Genome Annotation and Analysis (ORGAA) system.

The Oak Ridge National Lab (ORNL) Genome Annotation and Analysis (ORGAA) system is a complex software system developed over 10 years. In the ORGAA annotation system, pipelines are used to predict the locations of genes and functional RNA, assign functions to proteins, find functional protein domains, find repeats, characterize transporters and regulators, assign Enzyme Commission numbers (EC numbers), and create links to external databases.  A high level overview of ORGAA is shown in Figure 1. 

When we refer to a pipeline, we mean software systems that are implemented using the pipes and filters architecture [39].  A pipeline consists of (1) records – elements for storing data, (2) filters – elements for transforming data, (3) sources – a special type of filter that reads data from files or databases, (4) wrappers – software components that control the input and output to programs and program execution (e.g. 3 filters chained together, one filter for controlling the input, one filter for running the program, and one filter for reformatting the output), and (5) pipelines – software components that can be recursively assembled to construct larger systems.  A wrapper is also a pipeline.  Pipelines are stateless, and chained together in linear stages of execution.  Architecture patterns such as the pipes and filters architecture are used to manage the complexity of software systems. 

The ORNL annotation pipeline shares characteristics with the model organism databases that use Genetic Model Organism Database project components (GMOD) [23-25] to organize data.  However, it is different from systems focused primarily on eukaryotes because the annotators at ORNL have embedded the experience from annotating over 300 microbes.  Consequently, ORGAA is more accurately characterized as a general-purpose microbial annotation system.  

A new data representation has been developed herein for use in microbial genome annotation built on advancements in Semantic Web technology.  A microbial annotation system consists of algorithms for predicting genomic structure, functional features and creating relationships across databases, storage for algorithm results and monitoring and reporting subsystems.  

Our results and methods focus on: 

(1) Comparing the features of Semantic Web storage technologies to traditional annotation methods, 

(2) Discussing current limitations in data representation, 

(3) Illustrating how Semantic Web technologies can be used along side of conventional storage technologies, and 

(4) Illustrating how wrappers can be used to reformat the input and output of programs to produce data consistent with the Semantic Web and thus gain abilities not found in conventional systems.  

This provides a demonstration that a genome annotation system can be built on top of Semantic Web technologies and that these representations provide a layer of abstraction that has specific advantages in software development and are a natural fit for expressing the scientific concepts of annotation.

We intend to use the following terms from the emerging field of information and data quality to illustrate the advantages of this approach:

• Correctness:  Data is incorrect, when it contains logically inconsistent information or inconsistent formatting that leads to inconsistent results or causes downstream software processes to exhibit unexpected behaviour.

• Normalization: Data that is not normalized contains undesirable properties most notably, data modification abnormalities that lead to loss of data integrity (e.g. data correctness violations).

• Completeness:  A portion of the knowledge representation, A, is not complete if there exists accessible relevant information, B, that is disjoint from A.

### Overview of annotation data storage technologies

Annotation systems are built using several different data representations.  Most common are tab delimited file formats such as Gene Feature Format (GFF) [26].  Tab delimited file formats are easy to parse and easy to use in third party tools such as Microsoft Excel, however, column definitions are usually documented separately.  Free text or semi-structured files such as GenBank or EMBL files [27] are advantageous because they are the current standard for warehousing annotations and are human readable. However, this type of semi-structured data is resistant to change, because small changes in the standard tend to break parsers and legacy code.  Consequently, fields such as /note tend to get overloaded with useful information while product descriptions and gene names vary across annotation systems.  These variations make it extremely challenging to build general tools that are reliable.  Relational databases are flexible, robust, have a strong theoretical foundation and favourable runtime characteristics.  However schemas implemented in relational database systems tend to be complex and are usually owned by system maintainers.  It is not easy to implement a relational schema for every concept in biology because the focus of relational technology is on storage and retrieval of information and not on describing the information stored.  Also, it is difficult for domain experts (biologists) to construct and share relational schemas.

Other technical solutions are optimal for sharing data and performing queries across data repositories.  XML provides a viable alternative for sharing data across sites, but is difficult for biologists to read.  A more complete overview of relative advantages and disadvantages of data storage and exchange technologies is shown in Table 1.

**Table 1:** A comparison of five common data storage technologies currently deployed in annotation systems.

	Free Text	Tab/Line Delimited	XML	RDF/XML	Relational-DB
Description Logic (FOL)	NO	NO	NO	YES	NO
Ontology Standards	NO	NO	NO	YES	NO
Centralized/not scalable	NO	YES*	NO	NO	YES**
Human Readable	YES	YES	NO	YES	YES
Domain Expert Understandable	YES	YES	NO	YES	NO
Data Structure	NONE	Single Table	Tree	Graph	Relational Tables
Data Expectations	NONE	NONE	Schema - Constraints	Inference rules	Schema – Constraints
Native Format	Text	Text	Text	Text	Binary
Query Engine Language	Programmed by hand	Programmed by hand	Libraries available	SPARQL	SQL
Naming Standard	NO UNA****	NO UNA	NO UNA	NO UNA	UNA
Sequence Storage Solution	In Text	In Text	XML/Indexed	XML/Indexed	Indexed
CWA/OWA***	OWA	CWA	CWA	OWA	CWA
Search Speed (Worst Case)	NP-Hard	O(n)	O(n)	P	O(log n)
Update Speed (Worst Case)	NP-Hard	O(n)	O(n)	P	O(log n)
Conversion to Semantic	Data loss possible – done by hand	No data loss – done by hand	No data loss – done with robust libraries	-	No data loss –library usage and some added labeling by hand
Conversion from Semantic	No data loss	Data loss	Data loss	-	Data loss

Free text is used in repositories such as scientific journals.  Tab/Line delimited files are used in popular formats such as FASTA, GFF, and BLAST.  Tab/Line delimited files also constitutes the bulk of program output from most bioinformatics software.  Mature tools and sequence repositories such as GenBank support XML output.  Many OWL based ontology repositories exist for semantic data integration, however RDF/XML data is currently scarce. Relational databases typically do not provide direct access to the data, instead a programming interface is provided for access to the underlying database.  Free text is the most flexible, and also the least machine-readable.  Relational databases are the most formal structures with the fastest indexing and searching capabilities. Relational technology requires the greatest computational expertise investment while free text is the most natural.  XML and RDF/XML are designed for modification over time and in sharing data.   In the rows discussing search speed and update speed, O(log n), O(n), P and NP-Hard are computer science terms indicating a range of how fast a computer solution can be obtained to a particular problem.  P indicates a reasonable solution is possible in polynomial time, NP-Hard means that the solution space explodes relative to the input size.  NP-Hard problems are expected to not be solvable on a computer in reasonable time. O(log n), O(n), and P are all solvable efficiently on a computer.  In the rows discussing conversion to and from RDF/XML, Turtle, and other semantic aware data storage technologies, loss of information includes schema, constraints, data and formatting.  For example, to convert from a relational schema to tab-delimited files, information is lost because the schema, triggers and views are not representable using tab-delimited files.  So these columns are more than just data, they are data and descriptions surrounding the data for making logical conclusions and for executing computer codes in reasonable time.  In the conversion from free text to semantic standards, assumptions and domain expertise may be lost.

*Assuming all information is in one file.  If multiple files exist, then an indexing system needs to be developed to organize information.

**Relational databases are assumed to exist as a single installation on a powerful single resource.  New database technologies have lessened this restriction in recent years.

***CWA – Closed World Assumption, missing information treated as false. OWA – Open World Assumption, missing information treated as unknown.

****UNA Unique Name Assumption – Each individual has a single unique name.

A fundamental constraint of current annotation systems is that they are constructed with the explicit goal of submitting a sequence to GenBank/EMBL/DDBJ, the International Nucleotide Sequence Databases (INSD) [28].  Data warehousing has very different objectives than systems biology research and experimental data integration.  In practical terms, the objective of data submission to the INSD has resulted in sequence annotation systems that: (1) are based on text files that are computationally difficult to manipulate, and (2) cannot easily accept the expert knowledge possessed at the time of annotation, and (3) are difficult to merge with other datasets.  

Relational databases, free text files, tab-delimited files and XML have been adopted in annotation systems because each has desirable attributes.  Tab delimited files are easy to build Perl scripts around for rapid prototyping.  Relational technology is better suited to create robust and fast production systems with flexible query capability.  Next generation data models for biological annotation complement more traditional data storage techniques because they also provide a semantic layer for interoperability. 

RDF/XML and OWL have the potential to revolutionize annotation, because they share many of the favorable conventional storage techniques attributes, but also have new capabilities to represent data provenance and workflows, provide semantic data interoperability, allow for greater data transparency (accessibility by domain experts), and semantic consistency across systems.  These attributes will make it easier to store information that would otherwise be lost, create views on data (one such view is similar to the INSD representation), and perform logical inferences.

### Limitations to conventional representation technologies

As the annotation process proceeds, when additional algorithms are run, when databases are searched and results linked to the query, or when additional data is added to the system, information is created.  The order in which steps are allowed to occur and how the results from each of these steps are stored has significant impact on system behavior.  

Data stored in the system can be out of sync with data stored in other parts of the system.  As a simple example, consider updating a BLAST database.  A BLAST database is formatted from a collection of FASTA records.  BLAST scoring and results are dependent on the number and types or records in the FASTA file.  Results (e.g. e-values and best matches) prior to the update has the potential to be different than those obtained after the update.  Genomes annotated years ago commonly contain functional and positional annotation that was based on the best knowledge at the time but would be very different if the genome were annotated today.

Consider the following excerpts from two annotation files downloaded from GenBank representing results for two different annotation systems for the same gene in two closely related strains of* Escherichia  coli*:

/gene="dnaA"

/locus_tag="b3702"

/gene_synonym="ECK3694"

/gene_synonym="JW3679"

/function="putative regulator; DNA - replication, repair,

restriction/modification"

/note="DNA biosynthesis; initiation of chromosome

replication; can be transcription regulator;

GO_component: GO:0005737 - cytoplasm;

GO_process: GO:0006261 - DNA-dependent DNA replication"

/codon_start=1

/transl_table=11

/product="chromosomal replication initiator protein DnaA, DNA-binding transcriptional dual regulator"

/protein_id="NP_418157.1"

/db_xref="GI:16131570"

/db_xref="ASAP:ABE-0012103"

/db_xref="UniProtKB/Swiss-Prot:P03004"

/db_xref="ECOCYC:EG10235"

/db_xref="EcoGene:EG10235"

Example 1: *dnaA* from the annotation of *Escherichia coli* K12 substr. MG1655

/locus_tag="EcDH1_0001"

/product="chromosomal replication initiator protein DnaA" 

/inference="protein motif:TFAM:TIGR00362"

/note="TIGRFAM: chromosomal replication initiator protein DnaA" /note="PFAM: Chromosomal replication initiator DnaA domain; Chromosomal replication initiator DnaA" 

/note="SMART: AAA ATPase" 

/note="SPTR: A1AHN7 Chromosomal replication initiator protein dnaA" /note="KEGG: ssn:SSON_3652 chromosomal replication 

initiation protein  " 

/note="COGs: COG0593 ATPase involved in DNA replication initiation" /note="InterPro IPR001957:IPR003593:IPR013159:IPR013317" /codon_start=1 

/transl_table=11 

Example 2: *dnaA* from the annotation of *Escherichia coli* DH1 ATCC 33849

These annotations are from orthologous coding sequences for *DnaA* from different strains of *E. coli*.  They will be used to illustrate several known quality deficiencies in INSD formatted files.

1. **Omission errors.** These are a violation of the quality characteristic of completeness. Information available to the annotator at the moment of annotation is not stored in the INSD file, or it is stored in a way not understandable to other organizations (e.g. not linked to an ontology). Another type of omission is when dataset fields are sparsely populated, it may be possible to use an INSD tag, but it is not used because the INSD submission validation script causes an error, because the feature/qualifier is poorly documented, or because the annotation system does not support the functionality.  In the above example, not all tags are shared across annotations (the first example has /gene, /gene_synonym, /function, /protein_id, and /db_xref while the second has /inference).

2. **Version control errors:** These are violations of the quality characteristic of correctness. Under current INSD policy it is the responsibility of the scientist who submitted the annotation originally to maintain and update the annotation.  However, usually there is no incentive for updating annotation with small fixes that do not lead to  significant publications or other professional rewards.  Consequently many INSD annotations are out of sync with data in other databases or with other features unless the annotation is redone.

3. **Formatting inconsistencies.** These are violations of the quality characteristic of completeness. Often two communities develop terminology for the same thing independently.  These divergent syntaxes will both pass the INSD validation script.  However it will not be possible for a computer to determine that two fields mean the same thing.  Also, annotation systems may produce different syntaxes for the same biological concept over time.  In the above example ‘/note="SPTR: A1AHN7 Chromosomal replication initiator protein dnaA"’ indicates an external reference to DNAA_ECOLI (P03004, P78122, Q2M814) in SwissPro/UniProt/Tremble as does ‘/db_xref="UniProtKB/Swiss-Prot:P03004".  Another example is the slight inconsistency between the content in the  /product lines.

4. **Nonnormalized data.** These errors violate the principles of normalization, which attempt to reduce redundant data. Some datasets have repeated fields and superfluously duplicated field values derived from other sources.  An example is ‘GO_process: GO:0006261 - DNA-dependent DNA replication’.  Here  ‘DNA-dependent DNA replication’ is redundant data that is copied into the INSD file from GO for the purpose of improving readability.  Updating this information in the INSD file will cause it to be out of sync with GO.  Updates to the term in GO will cause it to be out of sync with what is in the INSD file unless both locations are simultaneously updated which usually is overlooked.

5. **Inconsistent data granularity.** These are violations of the quality characteristics of consistency and correctness.  Notes often contain many important bits of information in free text that are difficult to parse.  For example, /note="COGs: COG0593 ATPase involved in DNA replication initiation", contains a database reference (COG), a database ID (COG0593) and expert generated free text.  Fields in other records contain only a database reference.

6. **Incorrectly labeled records.** These are also violations of the quality characteristic of correctness. An example is a gene labeled with an incorrect function, or a group of genes with known function labeled as hypothetical.  As more individual scientists are allowed to annotate their own genomes it will become more difficult to regulate the usage of tags.  It will become harder to define usage, and meaning. This indicates a need for more education and training on information management skills, or an approach that allows more computer automation.  

7. **Poorly structured data.** These are violations of correct metadata description and relevance. In the example above, both annotations contain tags that are in free text.  This can not easily be compared by a computer.  Each annotation uses the /note tag differently.  In the first, /note tags are used to indicate function and to create unstructured links to gene ontology terms.  In the second, /note tags are used primarily to reference functional descriptions in other databases.  In both cases, bioinformaticians must build custom parser rules to fully take advantage of the information.  These parsers will not work on all of the files in the INSD, only those files formatted by the same annotation system.  In other descriptions, comparing text on the computer is nearly impossible.  Both usages illustrate qualifier tag overloading.

8. **Overloading of field function.**  Overloading, or using for more than one purpose, the function of qualifier tags (fields) in the database structure violates the quality characteristic of correctness.  It is often difficult to untangle the current function of a field if it has been used for many different functions.

9. **Overly rigid and inflexible standards:**  This makes adoption difficult. The three INSD collaborators must agree on common tags, which results in a slow rate of change.  Annotation systems have much more information available that is currently lost in the process of creating the INSD file.  Computational systems also have access to the series of events that created each of the qualifiers in the end file.  Currently, annotation systems are not allowed to use additional information or make information easier to parse with computers because the standard is based on consensus.  The result is that useful information is lost in the process of generating a genome annotation.  Some of this information is placed on web sites and is not stored in a machine readable way.

Semantic Web technologies can help resolve some of the inconsistencies between these two representations by linking the representations from multiple annotation groups into a single data representation.  This single representation could also contain information to record the history of information, and thus uncover the methods for obtaining the information.

### The semantic web technology stack applied to annotation

It is proposed here that next generation annotation systems should adhere to the following objectives: (1) Create less burdensome software requirements for downstream data users, (2) Provide a data model that is logically consistent with conventional methods, (3) Allow data integration from multiple sources, programs, groups or locations on the web, (4) Provide for a better understanding of how data is created, (5) Allow for a local level of standards that exceeds consensus standards, and thus permit technology advancement, and (6) Not create data loss or loss of functionality during data aggregation.  Meeting these objectives leads to an alternative model of genome annotation where each process adds additional information to the system, or creates links between information in the system or across systems.  RDF/XML and OWL are technologies designed to build systems of this type.  The OWL Web Language (OWL) is a specification for formally describing concepts and relationships between concepts.  This can be applied to the biological concepts or components of a software system.  RDF/XML [29]is a method for describing links from one data instances to another or from a data instance to a term (object) in an ontology.

OWL contains three constructs for describing data, the *Individual,* the* Class,* and *Properties.*  An *Individual,* or data instance is an element of data in the data repository.  A sequence is an example *Individual* (e.g. ‘attagacatccg’)  Each *Individual* has at least one *Class.AClass* is a set of individuals that share some property.  For example both *dnaA* and *nhaA* are members of the *Class*gene. Ontologies are constructed by nesting *Classes* in hierarchies.  This is accomplished through rdfs:subClassOf which states that one *Class* in the OWL ontology is a subclass of another class.  For example a protein_sequence is a subclass of a biological_sequence.  *Properties* can be used to state relationships, or triples, between individuals or from individuals to data values.  *Properties* can also be nested in classification hierarchies. Properties can also be constrained on both the domain and range.  For example a property hasFunctionalDescription can be constrained on the domain to objects that are genes, and it can be constrained on the range to objects that are functional descriptions or objects that are subclasses of functional descriptions.  Thus hasFunctionalDescription can be constrained to only provide relationships between instances that have the class gene and instances that have the class functionalDescription.

RDF/XML and OWL are built using triples.  Each triple contains a *subject (individual or class)*, a *predicate (property)* and an *object(individual or class)*.  Subjects, predicates and objects are all Uniform Resource Identifiers (URI) [30]. RDF/XML provides the specification for linking data instances to concepts (*Classes*) in OWL ontologies.  RDF triples can be used to link data instances to XML *datatypes* (e.g. ‘atacatccg isA xsd:string’), data instances to *individuals* (e.g. ‘dnaA owl:sameAs ECK3694’),  *properties* to other *properties* (e.g. ‘annotate:similarTo owl:SymmetricProperty sequence_ontology:homologous_to’), and data instances to *classes* (e.g. ‘dnaA isA sequence_ontology:gene’).  RDF triples allow for at least two important capabilities: (1) they allow constraints to be placed on data instances so that data is well formulated and consistent, and (2) they allow logical reasoning software to traverse ontology relationship hierarchies and thus answer complex questions.

In description logic terminology there is a fundamental modelling concept refer to as an **axiom** which is a logical statement that relate roles or concepts.  The set of such axioms that define concepts or terminology are referred to as a TBOX, and the set of axioms that define assertions or data are referred to as an ABOX.  A knowledge base consists of the combination of a TBOX and ABOX which can be served with RDF/XML being used as the ABOX, and OWL being used as the TBOX [22]. Annotation knowledgebase systems can be constructed such that they require that each algorithm and database search create RDF statements describing the provenance of data modifications and link discoveries in a semantically consistent way.

A knowledgebase constructed using OWL and RDF/XML has several advantages over conventional data representations:  

1. Semantics are explicitly stored in the system, making data unambiguous.

2. Because data is based on XML, data is easily stored and merged across parts of the system or across systems maintained by different groups.

3. Data is structured and can be queried in much the same way as in a relational database (using SPARQL [31]).

4. Standard algorithms (e.g. theorem provers [32-34]) can be used to analyze, query data repositories and make logical extensions to existing knowledge.

5. Data repositories of RDF/XML are inherently distributed over the web and thus processed in clustered computing environments and cloud computing.  

These advantages have yet to be realized because ontologies are integrated into data repositories in an ad-hoc manner.  In a semantic-aware knowledgebase system, semantic operations are not difficult to recover because they are imbedded in a data structure and not stored as free text, or embedded in programming logic. 

Below are some examples of how RDF/XML can be used to solve each of the problems with INSD files identified above.

1. **Omission errors.** Many omission errors of the type identified above can be corrected by linking annotations from different annotation groups.  Take the collection of all proteins with a significant alignment to *dnaA* from *E. coli* K12 as an example. RDF/XML is designed in such a way that it is possible to recover every qualifier that was ever associated to any of the proteins in this set.  Therefore omission errors can be greatly reduced by leveraging the strengths and experiences of different research groups and combining complementary datasets.  Simply mashing the tags together does not solve this because that does not constrain or classify tags, so there is no way to know how tags are related.  This would eventually result in a large set of redundant tags for each gene with no good method for filtering them.

2. **Version control errors:** RDF/XML is a web-based technology; it is not a static warehousing based technology.  Therefore, when an update happens on part of the data in the system, all of the records in the system reflect the change.  At any given moment, it is possible to construct an equivalent to the annotation file because it is just a view on the RDF data and not a flat file. 

3. **Formatting inconsistencies:**  RDF allows simple formatting inconsistencies such as the one described above to be resolved by querying the tag collection of highly related sequences and determining co-occurrence of terms.  Other formatting inconsistencies can be handled by associating explicit formatting rules to OWL classes and deprecating the use of free text in descriptions while enforcing the use of properly formatted data instances that belong to OWL classes.

4. **Nonnormalized data.**  RDF allows data to be normalized by explicitly removing redundant triples. 

5. **Inconsistent data granularity.** RDF requires that each relationship stated in a *predicate* links only two things.  Concepts linked in this way between well structured classes will not have multiple data instances encoded in a single relationship, unless users violate best practices.  

6. **Incorrectly labeled records.**  Because each instance must conform to a *class*, incorrectly labeled records can be identified by analyzing property labels of the same type in instances from superclasses/subclasses.  For example, imagine an instance of *dnaA* is incorrectly labeled as hypothetical.  An analysis of the associated GO *classes* of every near neighbor could be used to identify the inconsistency.  This sort of query is extremely labor intensive when ontology references are encoded as free text or not present.

7. **Poorly structured data.** Regardless of the annotation system or scientific group providing the data, if data is structured such that each instance must belong to a *class*, and that *class* is organized in an ontology, then data instances can be formatted to conform to the requirements of a *class.*

8. **Overloading of field function.**  RDF and OWL allow constraints to be placed on fields such that only instances of a particular type are allowed.  Instead of containing free text descriptions, tags can reference instances belonging to a specific* class.*  Because these instances belong to a specific* class*, it is possible to search* properties* that relate instances belonging to a particular type.  Therefore all known usages of a* properties* can be organized and described and new* properties* can be added in a logically consistent manner.

9. **Overly rigid and inflexible standards:** Instead of focusing on a globally accepted lowest common denominator standard, RDF and OWL allow individual system maintainers to focus on standards for solving particular problems.  This allows software systems to evolve at a different rate from data warehouses.

### Example of RDF/XML applied to annotation

XML has not been widely adopted by the bioinformatics community, perhaps because it is not human readable and not easily understood by domain expert biologists.  Here, we present TURTLE [35] showing how a human readable semantic-aware standard can be constructed as an alternative to standard GenBank files to overcome this limitation.  

gene   17489..18655

/gene="nhaA"

/gene_synonym="ECK0020"

/gene_synonym="ant"

/gene_synonym="antA"

/gene_synonym="JW0018"

/locus_tag="b0019"

CDS   17489..18655

/codon_start=1

/transl_table=11

/gene="nhaA"

/gene_synonym="ECK0020"

/gene_synonym="ant"

/gene_synonym="antA"

/gene_synonym="JW0018"

/locus_tag="b0019"

/product="sodium-proton antiporter"

/function="transport; Transport of small molecules:

Cations"

/note="Na+/H antiporter, pH dependent; GO_component:

GO:0009274 - peptidoglycan-based cell wall; GO_component:

GO:0019866 - organelle inner membrane; GO_process:

GO:0009268 - response to pH"

/db_xref="GOA:P13738"

/db_xref="InterPro:IPR004670"

/db_xref="PDB:1ZCD"

/db_xref="UniProtKB/Swiss-Prot:P13738"

/experiment="N-terminus verified by Edman degradation: PMID

8381959"

/protein_id="AAC73130.1"

/translation="MKHLHRFFSSDASGGIILIIAAILAMIMANSGATSGWYHDFLETP

VQLRVGSLEINKNMLLWINDALMAVFFLLVGLEVKRELMQGSLASLRQAAFPVIAAIGG

MIVPALLYLAFNYADPITREGWAIPAATDIAFALGVLALLGSRVPLALKIFLMALAIID

DLGAIIIIALFYTNDLSMASLGVAAVAIAVLAVLNLCGARRTGVYILVGVVLWTAVLKS

GVHATLAGVIVGFFIPLKEKHGRSPAKRLEHVLHPWVAYLILPLFAFANAGVSLQGVTL

DGLTSILPLGIIAGLLIGKPLGISLFCWLALRLKLAHLPEGTTYQQIMVVGILCGIGFT

MSIFIASLAFGSVDPELINWAKLGILVGSISSAVIGYSWLRVRLRPSV"

Example 3: The gene *nhaA* as it is stored in a traditional GenBank file.

Below is a TURTLE representation of the same record.  This example contains three *subjects*, gene:example_gene, CDS:exampleCDS, and experiment:exampleExperiment.  

@prefix an:  <http://compbio.ornl.gov/annotate.owl#> .

gene:example_gene

        a:hasStart integer:17489  ;

        a:hasEnd   integer:18655  ;

        a:hasStrand strand:+      ;

        a:obtainedFrom Prodigal V2.0

        a:hasGeneName string:nhaA ;

        a:hasGeneSynonym string:ECK0020 ;

        a:hasGeneSynonym string:ant ;

        a:hasGeneSynonym string:antA ;

        a:hasGeneSynonym string:JW0018 ;

        a:hasGeneSynonym string:sof ;

        a:hasLocusTag string:b0019 .

CDS:exampleCDS

        a:hasGene gene:example_gene ;

        a:codon_start integer:1   ;

        a:transl_table integer:11 ;

        a:hasLocusTag string:b0019 ;

        a:hasProductDescription string:sodium-proton antiporter ;

        a:hasFunctionDescription string:transport; Transport of small 

   molecules: Cations ;

        a:note string: Na+/H antiporter, pH dependent ;

        a:hasGO_component GO:0009274;

        GO:0009274 a:hasDescription peptidoglycan-based cell wall;

        a:hasGO_component GO:0019866;

        GO:0019866 a:hasDescription organelle inner membrane ;

        a:hasGO_process GO:0009268;

        GO:0009268 a:hasDescription response to pH ;

        a:hasdb_xref GOA:P13738  ;

        a:hasdb_xref InterPro:IPR004670 ;

        a:hasdb_xref PDB:1ZCD ;

        a:hasdb_xref UniProtKB/Swiss-Prot:P13738 ;

        a:hasExperiment experiment:exampleExperiment ;

        a:protein_id="AAC73130.1" ;

        a:hasTranslation

proteinSequence:"""MKHLHRFFSSDASGGIILIIAAILAMIMANSGATSGWYHDFLETP

VQLRVGSLEINKNMLLWINDALMAVFFLLVGLEVKRELMQGSLASLRQAAFPVIAAIGG

MIVPALLYLAFNYADPITREGWAIPAATDIAFALGVLALLGSRVPLALKIFLMALAIID

DLGAIIIIALFYTNDLSMASLGVAAVAIAVLAVLNLCGARRTGVYILVGVVLWTAVLKS

GVHATLAGVIVGFFIPLKEKHGRSPAKRLEHVLHPWVAYLILPLFAFANAGVSLQGVTL

DGLTSILPLGIIAGLLIGKPLGISLFCWLALRLKLAHLPEGTTYQQIMVVGILCGIGFT

MSIFIASLAFGSVDPELINWAKLGILVGSISSAVIGYSWLRVRLRPSV"""  .

experiment:exampleExperiment

        a:eve string:N-terminus verified by Edman degradation ;

	  a:hasEvidence edman degradation;

        a:hasdb_xref PMID:8381959 .

Example 4: The gene *nhaA* as it is stored in TURTLE, a RDF/XML equivalent.

The primary objective of this example is to illustrate how an RDF/XML representation can be displayed in a human readable way that is roughly equivalent to an INSD file; it may not be immediately obvious to the reader that a format conversion of this type is something significant.  However, it is significant in at least the following ways: (1) in it’s potential for correcting data representation errors such as omission errors, version control errors, formatting inconsistencies, nonnormalized data, inconsistent data granularity, poorly structured data, and overloading of field function, (2) in it’s capabilities for allowing for software and data abstraction and in alleviating overly rigid and inflexible standards, (3) in that constraints can now be placed on fields because well formed fields must be linked to terms in an ontology.

In the above example, the domain and range of each *predicate* is constrained such that each data instance is a member of a *class* and not just considered as free text.  In this example, gene:example_gene and CDS:exampleCDS are linked through the relationship CDS:exampleCDS a:hasGene gene:example_gene.  The *predicate*annotate:hasGene requires that the object referenced is of type gene.  These types of relationships can not be encoded in the INSD representation because tags in the INSD file are not ontology terms.  Higher level concepts such as what an a:gene is, and constraints on this data type are defined in the ORNL annotation OWL ontology, and, in this case, mapped to the ‘gene’ class in the sequence ontology (SO:0000704).  Constraints on the *predicates* in the example also exist in the annotation ontology.  

These constraints make it possible to normalize data, for example each gene_synonym in the GenBank example is repeated for both the CDS and the gene when in reality they only apply to the gene.  In RDF, because the CDS is associated with the gene, each synonym for the gene can also be associated with the CDS through the RDF graph.  

Omission errors can also be handled with this approach because RDF/XML allows us to merge data from any other data sources on the web such as KEGG [37] that already have RDF/XML accessible data [38]. This allows everything known about *nhaA* in any dataset to be linked to the annotation.  

Problems in the GenBank record with formatting inconsistencies, poorly structured data, inconsistent data granularity, and overloading of field function are all addressed in the TURTLE representation to a much greater degree.  The best example of this is the TURTLE alternative to the /note tag.  Instead of a free text description with GO identifiers sprinkled throughout, the TURTLE representation illustrates hard links to GO *classes* and free text descriptions of those terms.  This representation is easier to compute because the GO *classes* are easily distinguishable from free text by the computer. Another example is that, in the INSD file, experimental validation is represented in free text.  In the TURTLE representation annotators can represent both an evidence code that can be classified and placed in an evidence ontology (EV) [36], and they can link the data entry to the evidence method.  It is clear that this data representation scheme is better at handling the traditional problem of data loss than the INSD standard because statements do not have to pass a central regulatory body to be placed in the system.  Statements that would have been lost in the past can each be encoded relative to a local ontology.  At a later date, the meaning of these statements can be mapped to other ontologies as they develop, or the local ontology can be shared and become a standard.

RDF also addresses version control and normalization errors.  The TURTLE example above is actually an RDF result from a SPARQL query.  This separates the way the data is stored from the way the data is viewed.  In the case of the INSD file, the file contains the data.  This leads to data replication and loss of data integrity.  A much better approach is to have a file that contains references to other sources of information, as the referenced information improves, the annotation also improves.  Implementing a data cache can resolve performance issues.

The RDF example also imports relevant mapping to other ontologies (e.g. GO and SO).  This is done via importing the ORNL annotation ontology.  Concepts in the ORNL annotation ontology such as programs and databases can be defined locally independent of a central standards body.  An example of this is a:obtainedFrom Prodigal V2.0. This states that the region was obtained using the gene prediction program Prodigal.  The classification hierarchy that places Prodigal as a gene prediction program is stored in the ORNL annotation ontology, and is not deposited in INSD. This allows concepts to be declared and used rapidly local to the annotation system.  These concepts allow for a greater degree of abstraction.  Tool developers and data curators can declare concepts of importance to the task at hand without the burden of understanding the structure and function of annotation systems.

## Discussion

In the past, most work in bioinformatics focused on algorithms; however as more high-throughput experimental techniques become available, managing data complexity is an ever increasing challenge.  Bioinformatics systems are growing so quickly that it is no longer possible for a single source to fund data collection and analysis.  Better methods are needed to increase data quality and interoperability.  Advances can come from utilities that allow faster access, more intuitive access, more robust and sharable data structures, or the ability to provide new, increasingly detailed descriptions of data that are precise and machine readable even if manually contributed.  RDF/XML and OWL can be used in each of these ways, and have great potential to change the way data modelling is performed.  

It is important to recognize that RDF/XML, OWL, and other Semantic Web technologies are complementary** t**o traditional database technology, XML, programming languages (e.g. Perl) and other current techniques.  Resources built on these technologies need not be abandoned for the successful implementation of the Semantic Web; rather it is important that traditional technologies are linked in a logically consistent manner.  If this can be achieved, the Semantic Web will enable better software engineering practices, easier evaluation of system performance characteristics, and most importantly, a better understanding of the software processes and data used in systems biology.

An illustrative example of some of these advantages in annotation can be found in the current propagation of low quality product assignments.  Consider a protein sequence, *P*.  Assume that *P* has been determined biochemically to have a known function, *f.*  Annotation systems commonly copy the functional properties of *P* to putative proteins based on a sequence similarity cut-off.  Imagine that an annotation system encounters a hypothetical protein, *HP1*, that’s closest match is *P* and assigns *f* to *HP1.*  Now *HP1* is forever associated with *f* in INSD.  At a later date, another sequence annotation system performs a search for another hypothetical protein, *HP2* and assigns *f* as its function because the nearest neighbor to *HP2* is *HP1*.  This process continues until eventually a hypothetical protein, *HPX* which may have a function other than *f* is assigned the function *f.*  Semantic annotation allows more than the association of evidence codes to each annotation.  It also allows a trail of evidence (data provenance) to be constructed so that the methods and series of deductions leading to the functional assignment can be catalogued and ranked in a systematic way.  This allows for better characterization of algorithm performance and better understanding of systematic error.  More importantly, it allows such errors to be corrected.  It is possible that a system based on conventional storage technologies could implement this kind of data tracking in a local fashion; however, it is impossible for the inferences to be shared unless all users have nearly identical technology stacks.  The advantage of the Semantic Web approach is that it allows users with vastly different technology infrastructure to share and process inferences at a detailed level in a consistent and automated way.

## Conclusions

We have presented a conceptual framework for constructing a description logic and web standard based knowledgebase for genome annotation systems.  The core components of this system were introduced along with examples that illustrate the implementation details and advantages.  Some of the stated advantages include the ability to record a series of operations that are semantically consistent across groups and technologies, the ability to share data and semantics across research communities, greater data transparency (accessibility by domain experts), and logical consistency.

Semantic Web technology is still in its infancy and many important details need to be worked out to translate informatics research problems into production systems.  Although there are currently many implementations of core utilities, we do not yet know how to build systems that meet all of the stated expectations.  Large scale prototypes are needed to characterize performance issues and understand potential pitfalls.  

Our approach to creating a semantic annotation system will meet these challenges, but many underlying details are still being considered.  Combining multiple RDF data sources is a challenge because of the lack of available systems with such capabilities and researchers with sufficient expertise to build and demonstrate the benefits of such systems for biological research. When the web was first developed it was not immediately obvious how HTML formatting was better than text files.  Over time, as more web pages came online, the advantage of hyperlinks for navigating content became apparent.  RDF/XML and OWL create a standard for linking data.  This capability will increase in usefulness in a similar way as these methods are adopted and data accessibility increases.  The critical first step is to create RDF/XML data repositories that can be leveraged; annotation is a central resource in bioinformatics and is therefore an intuitive place to start.

For the most part, ontologies are not as useful as they could be because the analysis routines and data are not always linked in the most meaningful ways.  Ontologies developed with data that references and uses them will be more accurate, more robust, simpler to understand and more elegant.

## Methods

### System architecture

**Figure 2:**
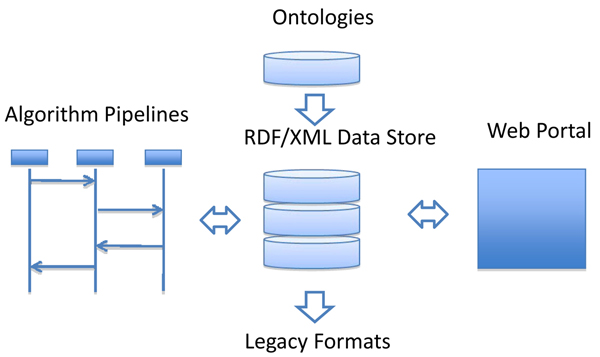
SAnoS System Architecture.  The open arrows represent the flow of data through the system.

The Semantic Annotation System (SAnoS) is a prototype system for creating genome annotations for the Semantic Web.  SAnoS builds on the ORGAA system.  It consists of pipelines for the discovery of genes, RNA and other functional features, database search tool wrappers (e.g. BLAST),  ontologies, and the capacity to create legacy formats (e.g. GenBank) from the Semantic Web data representation.  Figure 2 shows a high level overview of the functional aspects of the SAnoS architecture.  

At the center of the architecture is an RDF triple store (a database of RDF/XML triples, defined in the Results section) that stores the input and output from algorithm pipelines that create additional data instances and create links between datasets.  The triple store derives its data types from the ontologies available to the system.  Note that the triple store need not be one file, exist on one computer, or exist on one location on the internet. SAnoS algorithms for finding relationships in data sets are distributed, scalable, and require input and output to be RDF.  These pipelines are built using the pipes and filters architecture [39] and are built on top of the SAnoS core library.  The system can create an annotation view on demand.  This annotation can be exported to the web portal for viewing, or it can be converted to a legacy format through the legacy format conversion process.

### An ontology for annotation

Instead of assuming that one ontology could be built for the purposes of annotation, we instead assumed that several ontologies would be needed.  OWL representations of the Gene Ontology (GO), and the Sequence Ontology (SO) were obtained and used as a starting point.  Whenever possible, existing ontologies were leveraged when defining terms needed by the system.  In the construction of RDF/XML data stores this is accomplished via imports that reference the OWL document.  One imports an ontology into another to reuse the existing capabilities of the imported ontology in the new ontology.  Below is an example showing imports of relevant OWL ontologies in a TURTLE representation [35]. 

@prefix :        <http://compbio.ornl.gov/annotate.owl#> .

@prefix rdfs:    <http://www.w3.org/2000/01/rdf-schema#> .

@prefix owl2xml:  <http://www.w3.org/2006/12/owl2-xml#> .

@prefix xsd:     <http://www.w3.org/2001/XMLSchema#> .

@prefix owl:     <http://www.w3.org/2002/07/owl#> .

@prefix rdf:     <http://www.w3.org/1999/02/22-rdf-syntax-ns#> .

@prefix GO: <http://purl.org/obo/owl/GO> .

@prefix ECO: <http://purl.org/obo/ECO> .

@prefix SO: <http://purl.org/obo/owl/SO> .

@prefix CDAO: <http://cdao.cvs.sourceforge.net/viewvc/*checkout*/cdao/cdao/OWL/cdaov2.owl?revision=1.1> .

@prefix annotate:  <http://compbio.ornl.gov/annotate.owl#> .

Example 5: An example of import statements used in OWL.  In this example, the import statements are used to reference other ontologies in the current ontology.

Genome annotation pipelines. Oak Ridge National Lab’s (ORNL) as an example, contain many concepts that do not exist in standard Biological and Biomedical Ontologies (OBO).  For example, Prodigal [40] is a program used for determining the location and orientation of genes in a newly sequenced organism.  Statements dealing with data provenance such as ‘gene predictedBy Prodigal’ and  ‘Prodigal isVersion 2.0’ are possible with a locally controlled vocabulary.  We developed an annotation ontology to describe concepts and terms specific to the software process and terms unavailable in existing resources.  

**Figure 3:**
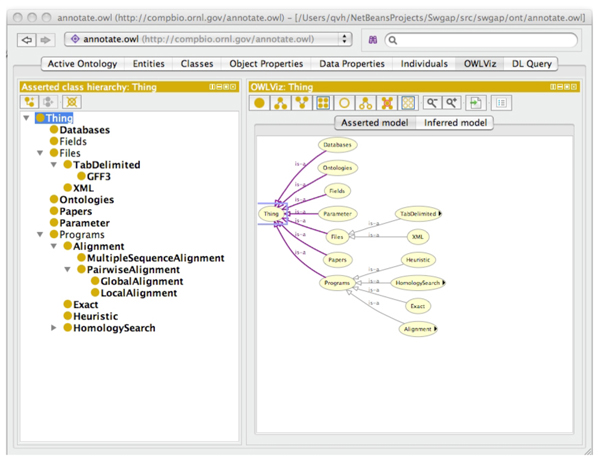
A simplified version of the ORNL annotation ontology edited in Protégé.

This ontology was developed and edited in Protégé [41].  Figure 3 shows an active session editing the ORNL annotation ontology. The OWL standard allows for *classes*, *properties*, *individuals*, and *datatypes*[15].  OWL provides structure for the underlying terminology used in describing data (a terminology box or TBOX in description logic [22]).  As other ontologies evolve, the OWL ‘owl:sameAs’ property is used to link semantic terms (concepts) across ontologies.

### Creating a wrapper for programs

To directly interact with the RDF/XML data store, an off the shelf program is wrapped with data formatting logic on the front end to query the RDF data store for data needed and to format the data into a form the program can understand.  Then the program is run on the reformatted data and creates an output.  The output from the program must also be converted to RDF/XML for storage in the data store.  

**Figure 4:**
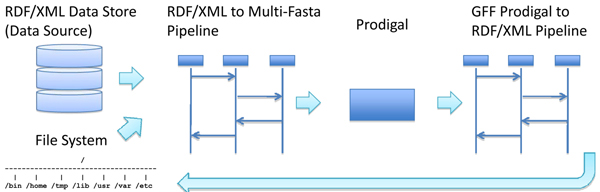
An example RDF pipeline for algorithm execution.

We will demonstrate the process here with the first step in the ORNL annotation pipeline, running the gene prediction software, Prodigal.  Figure 4 shows an implementation of a pipeline for running Prodigal.  Before Prodigal is run, raw sequence information is stored in the Data Source.   First, the data store is queried to obtain basic information (such as URIs) about sequences in the filesystem for processing.  Then Prodigal is run on the sequences.  This generates a Gene Feature Format GFF [26] file representing the predicted gene locations for each sequence in the sequence set.  A post processing pipeline converts the GFF file to RDF/XML representation.  This is accomplished by first querying the RDF store for triples directly related to sequences that the Prodigal application that was run on, and then associating the Prodigal predictions (in RDF) with the RDF results obtained from the data store.  This creates a new RDF file that references the annotation ontology and the sequence ontology.  This information, or a URI reference to it, can then be commited to the RDF store.

### Creating a RDF/XML data store

The RDF/XML data store consists of any set of RDF formatted files.  RDF formatted files reference OWL ontologies and other RDF files via URIs.  An RDF store is similar to a collection of web pages, each collection of statements imbedded in a page links other pages.  An arbitrary collection of RDF statements can be stored in a single relational database table, called an RDF triple store. Powerful tools for searching and manipulating RDF triple stores have emerged in recent years, most notable are query tools, and reasoning engines.  Jena [42] is a Semantic Web framework written in Java for the development of Semantic Web knowledge-bases.  The core Jena library contains utilities for manipulating and storing data models that are compatible with the Semantic Web.  Jena includes packages such as ARQ that implement a language processor for the SPARQL query language, SDB/TBD for implementing RDF storage comparable to relational database technology, and Pellet for OWL based reasoning over semantic resources.  The Jena technology suite was used to implement a prototype RDF-triple store for use by annotation pipelines.

### Accessing legacy information

**Figure 5:**
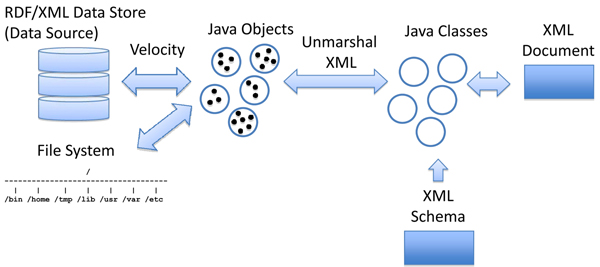
A Mechanism for translation between legacy formats and RDF/XML.

Despite the fact that XML/GenBank formatted documents contain many problems, large infrastructure is currently built on top of these standards.  To interface with legacy systems, we propose using the Java Architecture for XML Binding (JAXB) [44].  JAXB allows for seamless conversion from different flavours of XML.  This is accomplished by automatically constructing Java classes from an XML schema using a binding compiler, and then creating java objects with data from an XML file conforming to the schema.  Once the objects are instantiated, a reusable map is constructed that coordinates the translation from objects into an alternative XML schema.  For example, to construct Java objects from GenBank/EMBL files, first obtain the XML schema for GenBank/EMBL annotation [43].  Then, use the binding compiler, xjc, to derive java objects that are compatible with the GenBank/EMBL XML schema.  Third, translate files in GenBank/EMBL format to XML using the EMBL XML converter [43] or BioPerl [45]. Then use JAXB to unmarshal the content in the XML document into instances of java objects.  Finally construct the reusable JavaObject-XML mapping using the Velocity Template Engine [46] and use it to convert Java instances into RDF.  Process shown in Figure 5.  This process is reversible.  However, in translation of RDF/XML to INSD many semantically aware statements will be converted to free text.  In conversion of INSD to RDF/XML additional information will need to be added from other data sources and anthologies to fill in knowledge gaps and clarify inconsistencies.  

## Competing interests

The authors declare that they have no competing interests.

## Authors' contributions

ML and DQ outlined the problems with conventional storage technologies for annotation. TB and DQ designed the architecture for the system.  ML, DQ and RC provided the vision for the project.  DQ wrote the software and developed the prototypes.  DQ, ML and RC wrote the manuscript.  All authors approved the final version of the manuscript.
